# Volatiles from *Maruca vitrata* (Lepidoptera, Crambidae) host plants influence olfactory responses of the parasitoid *Therophilus javanus* (Hymenoptera, Braconidae, Agathidinae)

**DOI:** 10.1016/j.biocontrol.2018.11.002

**Published:** 2019-03

**Authors:** Djibril Aboubakar Souna, Aimé Hippolyte Bokonon-Ganta, Elie Ayitondji Dannon, Nazyhatou Imorou, Benjamin Agui, Antonino Cusumano, Ramasamy Srinivasan, Barry Robert Pittendrigh, Anne-Nathalie Volkoff, Manuele Tamò

**Affiliations:** a UMR DGIMI 1333 INRA, UM, Case Courrier 101, Place Eugène Bataillon, 34 095 Montpellier, France; b International Institute of Tropical Agriculture, Benin Research Station (IITA-Benin), 08 BP 0932 Tri Postal, Cotonou, Benin; c Department of Crop Production, Faculty of Agronomic Sciences (FSA), University of Abomey-Calavi (UAC), 03 BP 2819 Cotonou, Benin; d Michigan State University (MSU), Department of Entomology, East Lansing, USA; e World Vegetable Center (AVRDC), Tainan, Taiwan

**Keywords:** Biological control, Natural enemy, Olfaction, Attraction, Cowpea, Host plants

## Abstract

Plants damaged by herbivores are known to release odors attracting parasitoids. However, there is currently no information how leguminous plants damaged by the pod borer *Maruca vitrata* attract the exotic larval parasitoid *Therophilus javanus*, which was imported into Benin from the putative area of origin of the pod borer in tropical Asia for assessing its potential as a biological control agent. In this study, we used Y-tube olfactometer bioassays to investigate *T. javanus* response towards odors emitted by four *M. vitrata*-damaged host plants: cowpea *Vigna unguiculata*, the most important cultivated host, and the naturally occurring legumes *Lonchocarpus sericeus, Sesbania rostrata* and *Tephrosia platycarpa*. Olfactory attraction of *T. javanus* was influenced by the species of plant damaged by the pod borer. Moreover, odors released from *M. vitrata*-infested host plant organs (flowers and pods) were discriminated over non-infested organs in cowpea and *T. platycarpa*, respectively. These results are discussed in the context of the possible impact of *M. vitrata* host plants on *T. javanus* foraging activity and subsequent establishment in natural environments following experimental releases.

## Highlights

• The foraging behavior of *T. javanus* is influenced by pod borer *M. vitrata* host plants.
• Among wild host plant species, *T. javanus* is more attracted by odors of *T. platycarpa.*
• In cowpea, the cultivated host, infested pods are more attractive to *T. javanus* than infested flowers.

## 1 Introduction

*Maruca vitrata* (Fabricius) (Lepidoptera: Crambidae) is an insect pest of leguminous plants recorded on thirty-nine host plants species in Africa (Sharma et al., 1999, Arodokoun et al., 2003). In West Africa, cowpea *Vigna unguiculata* (L) Walp (Leguminosae) is the main cultivated host plant attacked by *M. vitrata* caterpillars and this pest can cause yield losses between 20 and 80% (Jackai and Daoust, 1986). Of the several host plants identified in Benin, the caterpillar primarily feeds on *Pterocarpus santalinoides* L'her. Ex De*, Pueraria phaseoloides* (Roxb.) Benth. and *Centrosema pubescens* Benth during the dry season, *Lonchocarpus sericeus* (Poir.) H.B. & K.*, L. cyanescens* (Schum. & Thonn.) Benth and *Sesbania rostrata* Bremek. & Oberm during the rainy season and *Tephrosia platycarpa* Guill. & Perr (all Leguminosae) during the short intermediate season (Arodokoun et al., 2003).

In view of the lack of host-specificity of local hymenopteran parasitoids attacking *M. vitrata* in West Africa (Arodokoun et al., 2006), and the confirmation of tropical Asia as the putative area of origin of the pod borer (Periasamy et al., 2015), Tamò et al. (2012) argued for the introduction of parasitoids from Asia into West Africa as a classical biological control approach. However, the first classical biological control candidate tested in West Africa, the larval parasitoid *Apanteles taragamae* Viereck (Hymenoptera: Braconidae), failed to establish substantial populations because of its inability to recognize major host plants of *M. vitrata* (Dannon, 2011)*.* In fact, the wasp was collected in Taiwan from *M. vitrata* caterpillars feeding on *Sesbania cannabina* (Retz.) Pers., but, surprisingly, it was not attracted by local *Sesbania* species in Benin (Dannon et al., 2012). Recent studies by Srinivasan et al. (2014) in tropical Asia have identified parasitoids more closely associated with and specific to *M. vitrata* feeding on yard-long bean (*V. unguiculata* subsp. *sesquipedalis*)*,* including *Therophilus javanus* (Bhat & Gupta) (Hymenoptera: Braconidae), a koinobiont, solitary, larval endoparasitoid introduced into Benin for preliminary assessment.

Parallel studies investigating the reproductive potential of *T. javanus* have evidenced its considerable fecundity and suitability for mass rearing (Aboubakar Souna et al., 2017). However, long-term reduction of *M. vitrata* populations depends mainly on how efficiently foraging *T. javanus* females will be able to detect and parasitize *M. vitrata* caterpillars throughout the cropping season and particularly during the offseason on alternative host plants.

It is well documented that odors emitted by plants can attract herbivorous insects and foraging parasitoids (Bruce et al., 2005, Wäckers, 2005). However, the odors may differently affect the behavior of the visiting insects (Pichersky and Gershenzon, 2002). For example, odors released from non-damaged plants can attract herbivore insects for feeding and/or reproduction but, when damaged, the plants will produce volatiles that can reduce herbivore oviposition and attract natural enemies (Bruce et al., 2005, Bruce and Pickett, 2011, Allmann et al., 2013). Herbivore-damaged plants are known to emit herbivore-induced plant volatiles (HIPVs), commonly used by parasitoids as host-searching cues (Vet and Dicke, 1992, Hare, 2011, Aartsma et al., 2017).

Host location behavior (and parasitism rate) in parasitoids can vary between different plant species of a given insect herbivore (Feng et al., 2015). With regard to olfactory responses to the pod borer host plants odors, there have been just a few studies carried out so far. Cowpea plants emitted volatiles attracting adult *M. vitrata* for feeding and oviposition (Wang et al., 2014, Feng et al., 2017). The pod borer larval parasitoid *A. taragamae* was attracted by odors released from caterpillar-infested cowpea flowers (Dannon et al., 2010). The objective of our study was therefore to document the olfactory response by foraging females of the exotic larval parasitoid *T. javanus* to odors of cowpea and three key alternative host plants *L. sericeus*, *S. rostrata* and *T. platycarpa*.

## 2 Materials and methods

### 2.1 Insects

Insect colonies were reared at the laboratories of the International Institute of Tropical Agriculture Benin (IITA-Benin) near Cotonou, Benin (12:12 L:D photoperiod; 26 °C ± 1.1 °C average temperature; 76% ± 7% relative humidity), with the methodology described in detail by Aboubakar Souna et al. (2017), and briefly summarized below.

Optimum egg production occurred in 4- to 5-day-old mated female *M. vitrata* (Jackai et al., 1990). During our experiment, five 4-day-old mated adult *M. vitrata* females were placed in transparent cylindrical plastic cups (3 cm diameter × 3.5 cm height) and kept for 24 h to allow for oviposition. Just prior to egg hatching, cups were opened and subsequently placed in cylindrical plastic containers (11 cm height × 16.5 cm diameter) containing sprouting cowpea seeds (Wetro et al, 2014) as a feeding substrate for *M. vitrata* caterpillars until pupation.

To rear the parasitoid, newly emerged males and females were kept together in a cage (15 cm on each side) for mating. After three days, 3-day-old *M. vitrata* caterpillars feeding on the sprouting cowpea seeds as described above were exposed to ten females *T. javanus* and reared until obtaining parasitoid pupae.

### 2.2 Host plants

Cowpea flowers and pods (at pod filling stage) were collected from unsprayed fields planted with the Benin local variety ‘Kpodji-guêguê’ at IITA-Benin (6°25′7.262″N 2°19′37.657″E). The flowers of the main wild host plants of *M. vitrata* (*S. rostrata, L. sericeus* and *T. platycarpa*) were collected from natural populations in the Zou Department in Benin, North of Cotonou (7°20′48.937″N 2°3′59.472″E).

### 2.3 Olfactometer setup

The response of 3-days-old naïve (without oviposition experience), mated females of *T. javanus* to plant volatiles was tested using a glass Y-tube olfactometer (Serbatoi Autoclavi, Type Elto, Vol. 50) as described by Dannon et al. (2010). Air was pumped through Teflon tubing, purified by passing through an active charcoal filter, and humidified through a jar containing distilled water. The internal diameter of the Y-tube measured 3.5 cm with an approximate wind speed in the olfactometer arms of 4 L/min. Each female parasitoid was transferred individually to the Y-tube, and the behavior of each individual was observed for 10 min as soon as it started moving. Females unable to move for more than 5 min at the release point, those moving but not entering in one of the Y-tube arms, as well as those not reaching the end of the arm were considered as non-responding. The positions of the odors sources were exchanged after testing five parasitoids to avoid bias by accidental asymmetry in the experimental setup. Each odor source was renewed after one hour. After testing each combination of odor sources, the olfactometer was cleaned with 75% ethanol followed by distilled water and then dried. All tests were carried out at the same laboratories condition as insect rearing.

#### 2.3.1 Response of *T. javanus* female to volatiles produced by *M. vitrata*-infested cowpea plant organs

Both *M. vitrata*-infested and uninfested cowpea flowers and pods were collected early in the morning (07:00–09:00 h), corresponding to the time of the day cowpea flowers are opening in the field (Ige et al., 2011). Flower were cut and kept separately in kraft paper bags to avoid odor contaminations. Prior to starting the experiments, collected flowers and pods were thoroughly observed under a stereomicroscope for detecting the presence of other insects, and to verify the instar of the larvae. In order to standardize the infestation conditions, only organs infested by *M. vitrata* second and third larval instars – corresponding to a feeding exposition of 3 days – (Okeyo-Owuor and Ochieng, 1981), at the rate of two caterpillars per organ, were considered for the experiment. For each odors source, we tested the effect induced by ten organs pooled together in glass jars connected to the olfactometer's arms.

Each of the following ten odors sources combinations were tested:

*Flowers*: (1) clean air versus uninfested flowers (80 females tested), (2) clean air versus caterpillar-infested flowers (160 females tested), (3) uninfested flowers versus caterpillar-infested flowers (160 females tested).

*Pods*: (4) clean air versus uninfested pods (80 females tested), (5) clean air versus caterpillar-infested pods (160 females tested), (6) uninfested pods versus caterpillar-infested pods (160 females tested).

*Both*: (7) uninfested pods versus uninfested flowers (80 females tested), (8) caterpillar-infested pods versus uninfested flowers (80 females tested), (9) uninfested pods versus caterpillar-infested flowers (80 females tested), and (10) caterpillar-infested pods versus caterpillar-infested flowers (80 females tested).

#### 2.3.2 Response of *T. javanus* females to volatiles produced by *M. vitrata*-infested wild host plants

Whole flower racemes were collected in early morning (07:00–09:00 h) from patches of wild host plants. The racemes of *S. rostrata, L. sericeus* and *T. platycarpa* were collected from Passagon (125 km, 2 h 30 min driving time), Massi (88 km, 1 h 50 min driving time), and Djidja (142 km, 3 h 35 min driving time) from the IITA-Benin station, respectively. Each fresh-cut healthy raceme was kept separately in kraft paper bags to avoid odors contaminations. To obtain infested flowers, two racemes of *S. rostrata*, *L. sericeus* and *T. platycarpa,* respectively, were placed separately in cylindrical plastic containers (9 cm diameter × 4.5 cm height) and artificially infested by introducing ten 3-day-old *M. vitrata* caterpillars in the containers for 24 h.

The attraction of *T. javanus* female parasitoids to (1) clean air versus uninfested flowers, (2) clean air versus caterpillar-infested flowers, and (3) uninfested flowers versus caterpillar-infested flowers were examined for each of the wild host plants. A total of sixty replicates (individual *T. javanus*) were assayed for each of the odors source combinations.

### 2.4 Statistical analysis

Adult female parasitoid choices were compared using a χ^2^ test to determine whether the observed distribution of responding wasps significantly diverged from a 50:50 distribution, which is expected if the wasps do not display any attraction toward the tested odors. The number of no-choice wasps was recorded but not included in the statistical analysis. The statistical software package R 3.3.2 (R Core Team, 2016) was used for all statistical analyses.

## 3 Results

### 3.1 Cowpea flower volatiles attraction

**Fig. 1:**
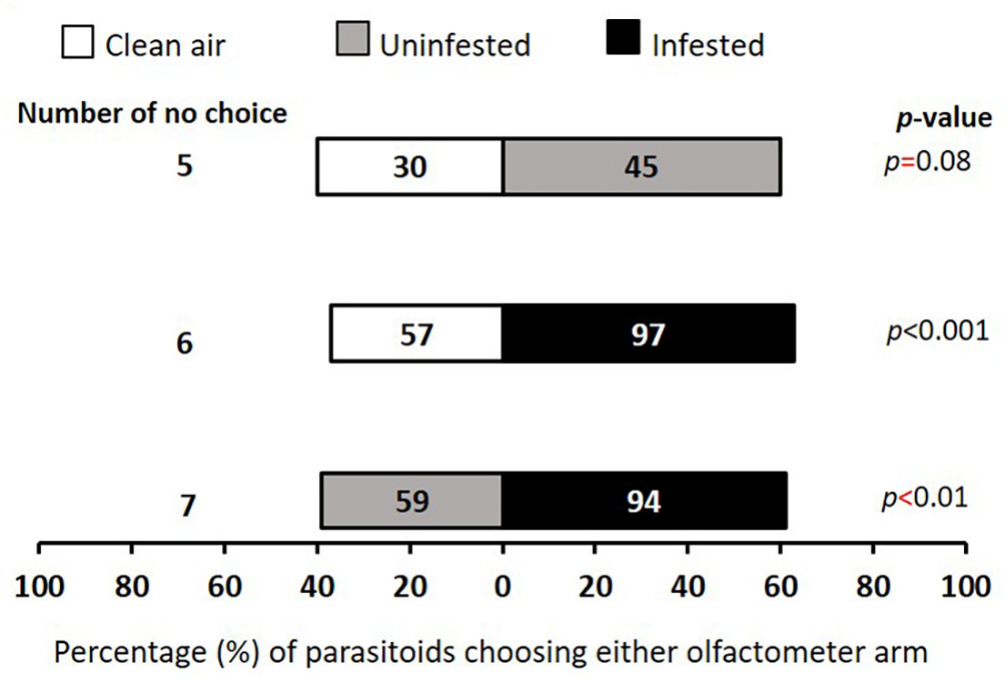
Response of female *T. javanus* when offered volatiles sources from cowpea flower in a Y-tube olfactometer. Numbers in the bars represent the total number of parasitoids that chose either olfactometer arm. Probabilities given to the right of bars are for the Chi-square test (*p* < 0.05).

The parasitoid did not discriminate between clean air and uninfested flowers (χ^2^ = 3, df = 1, *p* = 0.08). However, preference was displayed for caterpillar-infested flowers over clean air (χ^2^ = 10.39, df = 1, *p* < 0.001). Moreover, caterpillar-infested flowers were preferred over uninfested flowers (χ^2^ = 8.01, df = 1, *p* < 0.01) (Fig. 1).

### 3.2 Cowpea pod volatiles attraction

**Fig. 2:**
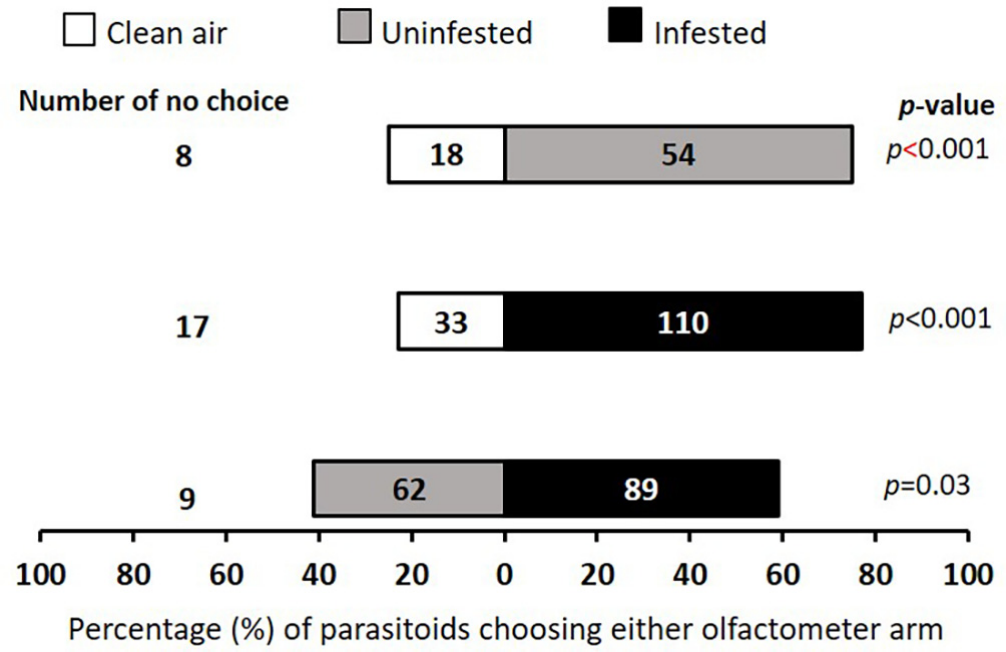
Response of female *T. javanus* when offered volatiles sources from cowpea pod in a Y-tube olfactometer. Numbers in the bars represent the total number of parasitoids that chose either olfactometer arm. Probabilities given to the right of bars are for the Chi-square test (*p* < 0.05).

The parasitoids showed significant preference to both uninfested pods (χ^2^ = 18, df = 1, *p* < 0.001) and infested pods (χ^2^ = 41.46, df = 1, *p* < 0.001) over clean air. They significantly preferred caterpillar-infested pods to uninfested pods (χ^2^ = 4.83, df = 1, *p* = 0.03) (Fig. 2).

### 3.3 Discrimination of cowpea pod and cowpea flower volatiles

**Fig. 3:**
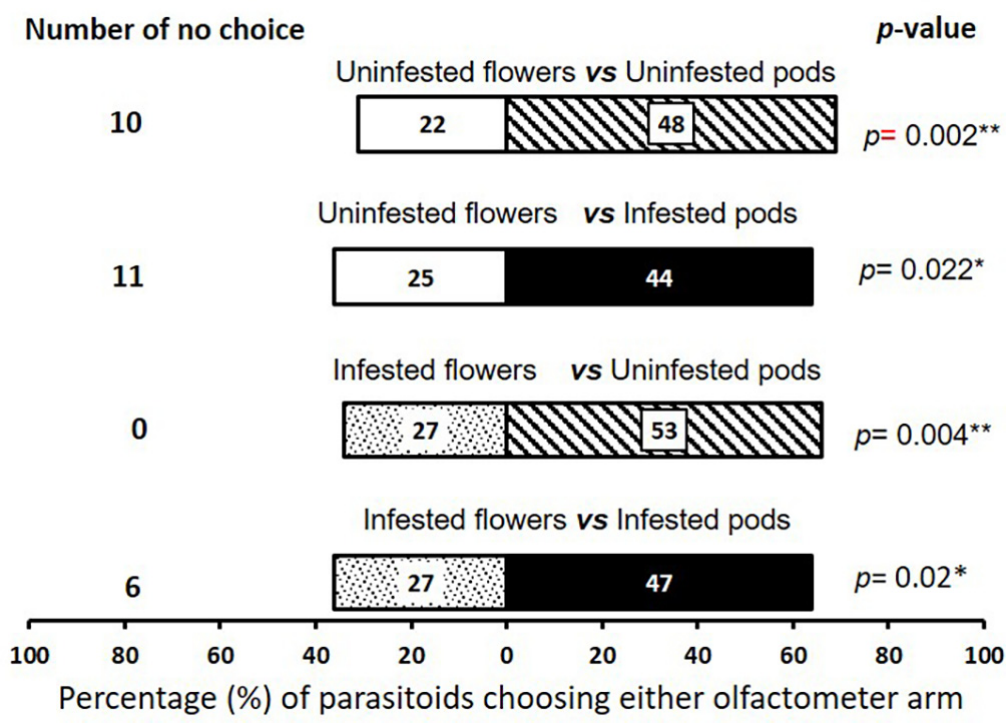
Response of female *T. javanus* when offered choices between cowpea flower and cowpea pod volatiles sources in a Y-tube olfactometer. Numbers in the bars represent the total number of parasitoids that chose either olfactometer arm. Probabilities given to the right of bars are for the Chi-square test (*p* < 0.05).

The wasps displayed a significant preference for uninfested cowpea pods over uninfested cowpea flowers (χ^2^ = 9.65, df = 1, *p* = 0.002) or caterpillar-infested flowers (χ^2^ = 8.45, df = 1, *p* = 0.004). Similarly, significant attraction to caterpillar-infested pods was observed over uninfested cowpea flowers (χ^2^ = 5.23, df = 1, *p* = 0.022) or caterpillar-infested flowers (χ^2^ = 5.40, df = 1, *p* = 0.02) (Fig. 3).

### 3.4 Wild host plant flowers volatiles attraction

**Fig. 4:**
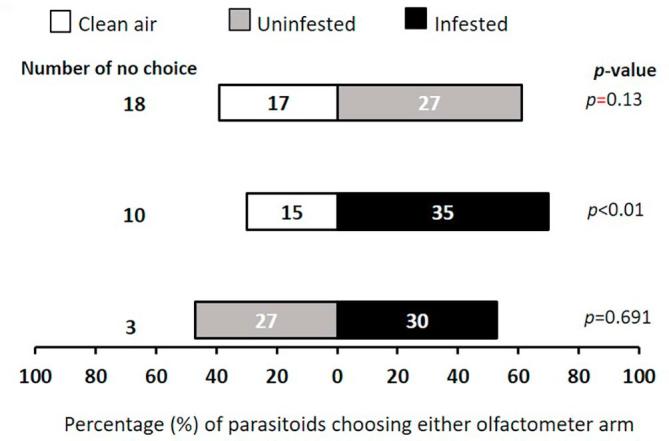
Response of female *T. javanus* when offered volatiles sources from *L. sericeus* flower in a Y-tube olfactometer. Numbers in the bars represent the total number of parasitoids that chose either olfactometer arm. Probabilities given to the right of bars are for the Chi-square test (*p* < 0.05).

**Fig. 5:**
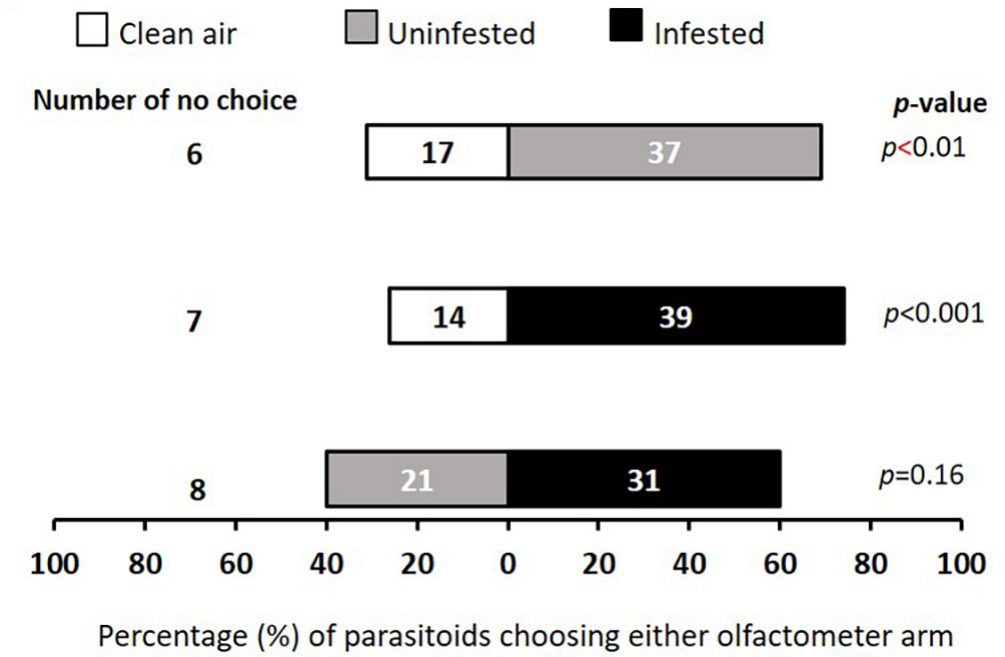
Response of female *T. javanus* when offered volatiles sources from *S. rostrata* flower in a Y-tube olfactometer. Numbers in the bars represent the total number of parasitoids that chose either olfactometer arm. Probabilities given to the right of bars are for the Chi-square test (*p* < 0.05).

**Fig. 6:**
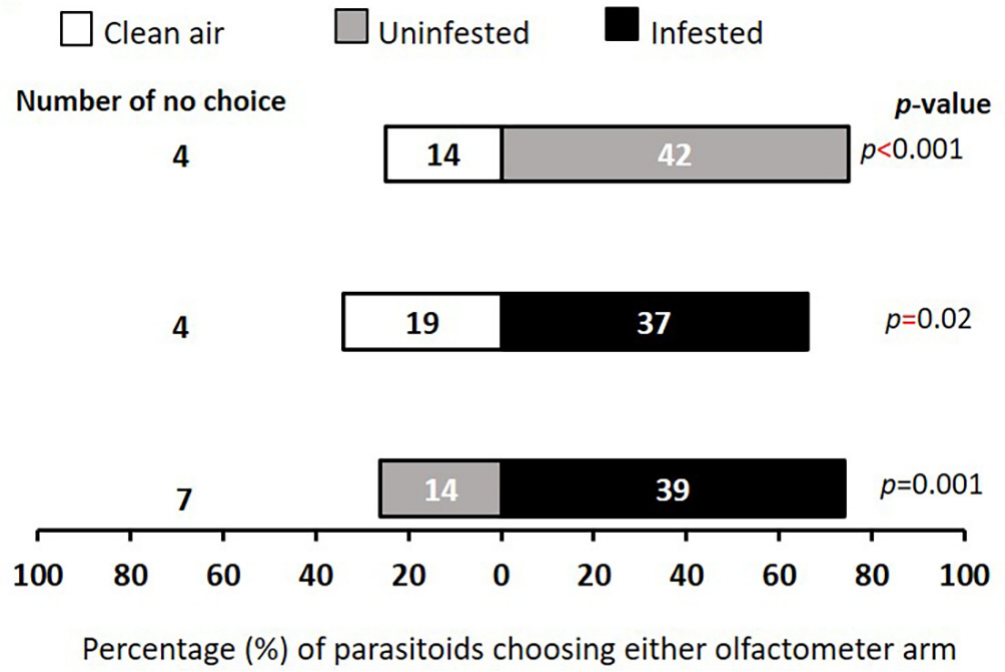
Response of female *T. javanus* when offered volatiles sources from *T. platycarpa* flower in a Y-tube olfactometer. Numbers in the bars represent the total number of parasitoids that chose either olfactometer arm. Probabilities given to the right of bars are for the Chi-square test (*p* < 0.05).

Generally, flowers were more attractive to the female *T. javanus* than clean air, although *L. sericeus* uninfested flowers were less preferred (Fig. 4). However, parasitoids did not display any significant preference when offered combination of odors sources from the same plant species, neither for flowers of *L. sericeus* (Fig. 4) nor for *S. rostrata* (Fig. 5). Uninfested flowers of *T. platycarpa* were less attractive than infested flowers of *T. platycarpa* (χ^2^ = 11.79, df = 1, *p* < 0.001) (Fig. 6).

## 4 Discussion

In this study, we showed that olfactory attraction of *T. javanus* was influenced by the species of plant damaged by the pod borer. In particular, odors released from *M. vitrata*-infested host plant organs (flowers and pods) were discriminated over non-infested cowpea organs. Odors can be produced by plants following herbivore damage for attracting natural enemies (Turlings and Wäckers, 2004, Arimura et al., 2009). Cowpea fully expanded trifoliate leaves are known to release novel odors compounds in response to herbivore infestation (Van Den Boom et al., 2004). Subsequent studies have reported that *Spodoptera littoralis* (Boisduval) (Lepidoptera: Noctuidae) caterpillar infested cowpea plant released specifics volatiles attracting females of the parasitoids *Campoletis sonorensis* (Cameron) (Hymenoptera, Ichneumonidae), *Microplitis rufiventris* Kokujev (Hymenoptera: Braconidae) and *Cotesia marginiventris* (Cresson) (Hymenoptera: Braconidae) (D’Alessandro and Turlings, 2005, Sobhy et al., 2018). However, there have been only a few studies investigating *M. vitrata*-infested cowpea flowers releasing odors to attract parasitoids (Dannon et al., 2010). In our work, *T. javanus* females were attracted both by odors of infested cowpea flowers and infested pods. However, the parasitoid preferred caterpillar-infested cowpea pods over caterpillar-infested flowers, suggesting that odors released from cowpea pods may be more detectable to the female parasitoids*.* According to Turlings et al. (1993), the quality of odors released by the plant attacked by the same caterpillar species can vary depending on the organs infested. Therefore, the discrimination of the pod odors observed in the wasps could be due to difference in herbivore-induced plant volatile composition.

We observed that the female parasitoids were able to distinguish undamaged cowpea pods from infested cowpea flowers. Therefore, we cannot completely discard the hypothesis that even undamaged cowpea pods may release odors that can be used as an attractive signal cue for the female *T. javanus*. For example, Kigathi et al. (2009), identified several herbivore-induced volatiles released at low level from undamaged forage legume plants, *Trifolium pratense* L., (Leguminosae). The fact the female parasitoid discriminated undamaged cowpea pods also might be attributed to the odors composition variability between infested cowpea flowers and undamaged pods. To date, several studies have identified whole cowpea plant (Lwande et al., 1989, Bendera et al., 2015, Zhou et al., 2015, Sobhy et al., 2018), leaf (Van Den Boom et al., 2004) and floral volatiles (Andargie et al., 2014, Wang et al., 2014, Feng et al., 2017), but none has ever investigated cowpea pod volatiles. However, it has been shown that volatiles varied quantitatively at different phenological stages of another leguminous crop, pea (*Pisum sativum* L.). The pea pod releases volatiles which are more attractive to the pea weevil (*Bruchus pisorum* L.) (Coleoptera: Bruchidae) than flower volatiles or volatiles from whole plants (Ceballos et al., 2015). But why would *T. javanus* be more attracted to pod volatiles? Female *T. javanus* may be able to adjust host localization strategies and choose host microhabitat that can enhance her offspring survival probability. Phytophagous insects have developed different feeding strategies to escape natural enemies (Connor and Taverner, 1997), including concealed feeding habitat that may limit predation and parasitism risks (Tschanz et al., 2005). *Maruca vitrata* caterpillars predominantly feeds inside plant organs. Feeding starts from the green, unopened flowers, with growing caterpillars moving to older flowers and ultimately pods where it completes its development (Bailey, 2007, Jayasinghe et al., 2015). Feeding inside growing cowpea pods might incur less mortality risks (e.g. by predation) than if moving between two flowers or from flower to pods. The higher protection conferred by a concealed feeding habitat applies to both non-parasitized and parasitized hosts. Mortality of parasitoid offspring is closely related to the parasitized host mortality (Fritz, 1982). Hence, foraging parasitoids have adopted strategies such as the innate preference for odors released from host microhabitat, to enable them to choose suitable hosts that minimize offspring mortality during immature stages development (Vet and Dicke, 1992, Hedlund et al., 1996) or developed long ovipositors to probe and parasitize concealed host (Sharkey, 1992). We can, therefore, hypothesize that *T. javanus* may be attracted to volatiles released from caterpillar-infested pods as host microhabitat, as a strategy to minimize mortality risk of its offspring.

Floral odors compositions generally vary between closely related species (Knudsen et al., 2006). Undamaged leaves and flowers of the peabush *S. cannabina* were reported to release odors attracting the female parasitoid *A. taragamae* females when tested against clean air (Dannon et al., 2010). In our work, among the three plant species tested (*L. sericeus*, *S. rostrata*, and *T. platycarpa*.), only infested flowers of *T. platycarpa* exhibited significant attraction to female parasitoids when tested against uninfested flowers of the same plant. These observations are in agreement with previous studies indicating that different host plants can emit specific volatiles signaling the presence of herbivores (Dicke et al., 2003, Turlings and Wäckers, 2004). The discrimination of infested vs. uninfested flowers of *T. platycarpa* may be due to specific volatiles released by the plant in response to herbivore infestation.

The long-term goal of this research was to determine the parasitism competence of the exotic wasp *T. javanus* foraging for the pod borer *M. vitrata* in a new environment in Africa. Our studies have showed that cowpea pods fed upon by the pod borer caterpillars are attractive to the parasitoid and these are encouraging news. However, we also observed that not all *M. vitrata* wild host plants detached flowers were able to emit volatiles attracting the foraging parasitoids, calling for future bioassays to be carried out in more natural settings (Ballhorn and Kautz, 2013). In fact, several factors can influence odors compositions and releasing intensity in plants (Paré and Tumlinson, 1999): the degree of the biotic stresses such as herbivores damage (Niinemets et al., 2013), and abiotic stress such as light intensity, time of year, water stress, and nutrient availability (Takabayashi et al., 1994, Becker et al., 2015). Therefore, complementary investigations of the parasitoid attraction to different host plants (focusing on flowers and pods) in natural environments could lead to a more consistent assessment of the host finding behavior of foraging parasitoids (Wäschke et al., 2014). On the other hand, the ability of a parasitoid to find the appropriate host habitat can be influenced by varying quantities and/or qualities of released volatiles, which can differ among plants species exposed to the feeding activity of the same polyphagous herbivore (Veterans, 1992, Becker et al., 2015), such as the cowpea pod borer. To overcome these obstacles in host habitat recognition, and enhance the host finding efficiency of foraging parasitoids, novel approaches are targeting the biosynthesis of specific and effective HIPVs (Peñaflor and Bento, 2013, Sobhy et al., 2018) that can be applied on the target crop for enhancing parasitoid recruitment (James et al., 2005). Hence, further investigations should attempt to identify specific volatile compounds (and proportions of compounds) emitted by cowpea and other wild host plants, and their different organs attacked by caterpillars of the pod borer, in order to assess the quantitative and qualitative responses of candidate biological control agents such as *T. javanus* to these HIPVs, with the long-term goal to re-engineer the chemical ecology dominating the complex tritrophic interactions between the crop, herbivores and their natural enemies, and make the latter more competitive.

## Author statement

Djibril Aboubakar Souna: conceptualization, methodology, data collection and curation, writing-original draft preparation.

Aimé Hippolyte Bokonon-Ganta: conceptualization, methodology, supervision, writing-reviewing and editing.

Elie Ayitondji Dannon: methodology, data curation, writing-original draft preparation.

Nazyhatou Imorou: data collection, writing-original draft preparation.

Benjamin Agui: data collection, writing-original draft preparation.

Antonino Cusumano: methodology, data curation, writing-original draft preparation.

Ramasamy Srinivasan: writing-reviewing and editing.

Barry Robert Pittendrigh: writing-reviewing and editing, resource mobilization.

Anne-Nathalie Volkoff: conceptualization, methodology, supervision, writing-reviewing and editing, resource mobilization.

Manuele Tamò: conceptualization, methodology, supervision, writing-reviewing and editing, resource mobilization.
